# Extracellular Matrix Stiffness: Mechanotransduction and Mechanobiological Response-Driven Strategies for Biomedical Applications Targeting Fibroblast Inflammation

**DOI:** 10.3390/polym17060822

**Published:** 2025-03-20

**Authors:** Watcharaphol Tiskratok, Nontawat Chuinsiri, Phoonsuk Limraksasin, Maythwe Kyawsoewin, Paiboon Jitprasertwong

**Affiliations:** 1 Institute of Dentistry, Suranaree University of Technology, Nakhon Ratchasima 30000, Thailand; cnontawat@sut.ac.th (N.C.); paiboonj@sut.ac.th (P.J.); 2 Oral Health Centre, Suranaree University of Technology Hospital, Nakhon Ratchasima 30000, Thailand; 3 Center of Excellence for Dental Stem Cell Biology, Department of Anatomy, Faculty of Dentistry, Chulalongkorn University, Bangkok 10330, Thailand; phoonsuk.l@chula.ac.th (P.L.); leomaythwe@gmail.com (M.K.)

**Keywords:** substrate stiffness, extracellular matrix, polydimethylsiloxane, hydrogel, fibroblasts, inflammatory responses

## Abstract

The extracellular matrix (ECM) is a dynamic network providing mechanical and biochemical cues that regulate cellular behavior. ECM stiffness critically influences fibroblasts, the primary ECM producers, particularly in inflammation and fibrosis. This review explores the role of ECM stiffness in fibroblast-driven inflammation and tissue remodeling, focusing on the physicochemical and biological mechanisms involved. Engineered materials, hydrogels, and polydimethylsiloxane (PDMS) are highlighted for replicating tissue-specific stiffness, enabling precise control over cell–matrix interactions. The surface functionalization of substrate materials, including collagen, polydopamine, and fibronectin, enhances bioactivity and fibroblast adhesion. Key mechanotransduction pathways, such as integrin signaling and YAP/TAZ activation, are related to regulating fibroblast behaviors and inflammatory responses. The role of fibroblasts in driving chronic inflammatory diseases emphasizes their therapeutic potentials. Advances in ECM-modifying strategies, including tunable biomaterials and hydrogel-based therapies, are explored for applications in tissue engineering, drug delivery, anti-inflammatory treatments, and diagnostic tools for the accurate diagnosis and prognosis of ECM stiffness-related inflammatory diseases. This review integrates mechanobiology with biomedical innovations, providing a comprehensive prognosis of fibroblast responses to ECM stiffness and outlining future directions for targeted therapies.

## 1. Introduction

The extracellular matrix (ECM) is a dynamic, three-dimensional network that provides the mechanical support and biochemical signals essential for the regulation of critical cellular processes, including proliferation, differentiation, and migration [1]. Tissue-specific stiffness varies widely, from soft tissue with low stiffness to rigid tissue with high stiffness, and these ECM stiffness levels are essential for maintaining tissue homeostasis and function [2]. Furthermore, ECM stiffness has emerged as a critical determinant of cell behavior, particularly in the context of inflammation and tissue remodeling [3,4]. Fibroblasts, the primary producers of ECM components, are highly sensitive to mechanical cues and are pivotal players in the interplay between ECM stiffness and inflammatory responses [5]. Recent advances in material science and mechanobiology have opened new avenues for modulating ECM stiffness to influence fibroblast behavior, with promising implications for biomedical applications [6,7]. Understanding how ECM stiffness influences cellular behavior is a cornerstone of biomedical research, enabling the study of cell–matrix interactions in controlled environments and offering insights into tissue engineering, disease modeling, and mechanobiology [8].

Regarding the biomaterials needed to replicate ECM stiffness, polydimethylsiloxane (PDMS) and hydrogels have emerged as leading candidates, owing to their tunable mechanical properties, biocompatibility, and versatility [9,10]. PDMS, a silicone-based elastomer, is widely used to mimic the stiffness of various tissue types [10]. Hydrogels, on the other hand, are hydrophilic polymer networks that excel in replicating the ECM because of their high water retention, tunable mechanical properties, and capacity for biofunctionalization [11]. Both materials are used for studying cell–matrix interactions, particularly in models involving fibroblast behavior and inflammatory responses. Furthermore, the development of advanced biomaterials to mimic ECM stiffness is a concern. Surface functionalization is another crucial aspect of enhancing the bioactivity of these biomaterials; collagen, oxygen plasma, and polydopamine enhance material bioactivity, enabling precise control over cell–matrix interactions [12,13].

The mechanical properties of the ECM are not only critical for maintaining tissue integrity, but also play a significant role in disease progression [14]. Previous studies demonstrated that increased ECM stiffness is a hallmark of fibrotic tissues, which are characterized by excessive deposition of ECM components such as collagen. This heightened stiffness enhances fibroblast activation and proliferation, leading to a positive feedback loop that perpetuates inflammation and fibrosis [15]. Mechanotransduction pathways, including integrins, focal adhesion kinase (FAK), Rho-associated kinase (ROCK), and Yes-associated protein (YAP), are central to these processes [16]. These pathways convert mechanical signals into biochemical responses, regulating gene expression and cellular behavior in response to changes in ECM stiffness. These mechanisms are essential for developing therapeutic strategies aimed at modulating ECM properties to mitigate inflammation and fibrosis [8]. In addition to their role in fibrosis, ECM stiffness and fibroblast behavior are implicated in a wide range of inflammatory conditions across various tissues, including cardiac, pulmonary, skin, intestinal, skeletal muscle, and periodontal tissues. For cardiac fibrosis, increased ECM stiffness promotes the secretion of pro-inflammatory cytokines, exacerbating inflammation and tissue remodeling [17]. Similarly, in pulmonary fibrosis, fibroblasts exposed to stiffer substrates exhibit enhanced secretion of pro-inflammatory cytokines, such as IL-1β and fibrotic expression, contributing to disease progression [18]. These findings highlight the importance of ECM stiffness as a regulator of fibroblast behavior and inflammatory responses in both physiological and pathological contexts.

The development of advanced substrate stiffness biomaterials is beneficial for studying the role of ECM stiffness in disease modeling and therapeutic applications [19,20]. These biomaterials enable the precise control of mechanical properties to replicate tissue-specific stiffness and investigate cellular responses under controlled conditions. Furthermore, other strategies, including enzymatic ECM degradation, biomaterial engineering, and hydrogel-based therapies, offer promising approaches for modifying ECM stiffness and treating ECM stiffness-related diseases [2,21]. Non-invasive imaging techniques, such as magnetic resonance elastography (MRE) and ultrasound elastography, have also emerged as valuable diagnostic tools for assessing tissue stiffness and monitoring disease progression [22,23].

Therefore, this review aims to provide a comprehensive overview of the role of ECM stiffness in regulating fibroblast behavior and inflammatory responses. It highlights the importance of synthetic materials such as PDMS and hydrogels in replicating tissue-specific stiffness and studying cell–matrix interactions. Additionally, it explores the mechanotransduction pathways involved in fibroblast activation and the implications of ECM stiffness in various inflammatory conditions. Finally, it discusses the potential of ECM-modifying strategies and advanced imaging techniques for developing innovative therapeutic interventions [24]. By elucidating the interplay between ECM stiffness, fibroblast behavior, and inflammation, this review provides a foundation for the development of innovative therapeutic strategies targeting fibroblast inflammation diseases. Furthermore, this review further highlights how ECM stiffness modulation contributes to biomedical innovations, including tissue engineering, using stiffness-tunable scaffolds and hydrogels to mimic healthy and pathological tissue mechanics. Additionally, drug delivery systems target inflammatory pathways. ECM-modifying strategies, such as soft substrates for disease modeling and stiffness-responsive therapies, mitigate fibrosis and inflammation.

## 2. Biomaterials for Mimicking Extracellular Matrix Stiffness in Tissue

The ECM is a complex network of proteins and polysaccharides that provides structural support and regulates cellular behavior in tissues. Its mechanical properties, particularly stiffness, play a pivotal role in influencing cell migration, proliferation, differentiation, and inflammation [25,26]. Tissue-specific stiffness varies widely, from soft brain tissue with low stiffness to rigid bone tissue with high stiffness, and these variations are critical for maintaining tissue homeostasis and function [27].

Developing the mechanical properties of biomaterials that mimic ECM stiffness in vitro has become a cornerstone of biomedical research, enabling the study of cell–matrix interactions in controlled environments. Synthetic materials that mimic ECM stiffness are essential for tissue engineering, disease modeling, and mechanobiology mechanisms. Among these materials, polydimethylsiloxane (PDMS) and hydrogels have emerged as leading candidates, owing to their tunable mechanical properties, biocompatibility, and versatility. These biomaterials provide effective platforms for investigating how the mechanical cues from ECM stiffness influence cellular behavior, providing critical insights into tissue development, disease progression, and therapeutic interventions.

### 2.1. Polydimethylsiloxane (PDMS)

PDMS consists of a three-dimensional (3D) polymer network created through the thermal curing of its liquid prepolymer components. These components include an elastomer base, such as a vinyl-terminated siloxane, and a crosslinking reagent, such as a hydrogen-terminated siloxane, in the presence of a platinum catalyst [28]. PDMS is a component within the well-known organosilicon group of silicones. It is a highly versatile silicone-based elastomer that is a subclass of polymeric materials, widely used in biomedical research to replicate ECM stiffness in various tissue types [29]. Its tunable mechanical properties, biocompatibility, and ease of fabrication have established PDMS as a widely used material in the investigation of cell–matrix interactions, particularly in models focused on fibroblast behavior and inflammatory responses [30,31]. This review provides an overview of the distinctive properties of PDMS, its application in mimicking tissue stiffness, and its role in cellular responses to substrate stiffness. Additionally, we present an integrated protocol for fabricating PDMS substrates with tailored stiffness, offering insights into how it can be utilized to investigate fibroblast inflammatory responses.

The ability of PDMS to replicate a wide range of tissue stiffnesses is a significant advantage. By varying the crosslinking ratio of PDMS, previous studies have modified substrate stiffness by mimicking the mechanical properties of brain tissue and more rigid tissues such as bone [32]. This adaptability property of PDMS makes it an ideal material for fabricating substrates that replicate the wide range of ECM stiffnesses found in human tissue. Such substrates facilitate the study of stiffness-dependent cellular behaviors, including migration, proliferation, and differentiation, particularly regarding fibroblast activity and inflammatory responses [20].

The PDMS structure features a backbone of alternating silicon and oxygen atoms, with methyl groups attached to the silicon atoms. This distinctive structure provides PDMS with flexibility, chemical inertness, and hydrophobic properties, which have facilitated its broad utilization in industrial and biomedical applications [33]. PDMS is valued for its ease of processing, ability to be functionalized, and capacity to replicate complex microstructures. This versatility has confirmed its role as a foundational material in tissue engineering and microfabrication, creating an ideal platform for determining the impact of substrate stiffness on cellular responses in inflammation and fibrosis [34,35,36].

The chemical structure of PDMS, represented as [Si(CH_3_)_2_O]_n_ units, features a siloxane backbone composed of alternating silicon and oxygen atoms, which imparts exceptional stability and resistance to chemical degradation. The silicon–oxygen bond (Si-O-Si) contributes low surface energy, resulting in hydrophobicity. This distinctive structure endows PDMS with several key properties. The Si-O-Si bonds are relatively weak compared to those in organic polymers, resulting in a highly flexible and extensible polymer chain, which is crucial for applications that mimic the compliance of natural tissues [37]. Furthermore, the methyl groups (CH_3_) attached to the silicon atoms enhance the chemical inertness of PDMS, reducing its reactivity with other substances and ensuring biocompatibility. These properties make PDMS highly suitable for applications in medical devices and tissue engineering [38]. Additionally, crosslinking reactions incorporating functional groups such as phenyl and vinyl can substantially modify the material’s properties, facilitating customization for a wide range of applications [39]. PDMS exhibits remarkable thermal stability and maintenance properties. Furthermore, the mechanical properties of PDMS are influenced by both heating temperature and time. At lower temperatures, the heating time has little effect on mechanical strength. However, as the temperature increases, its mechanical strength decreases, especially with prolonged heating above 200 °C [40]. Interestingly, previous studies have reported that the stiffness of soft PDMS can be as low as 5 kPa [41], while stiff PDMS formulations can achieve stiffness values of up to 10 MPa [42]. Therefore, PDMS is suitable for temperature-sensitive applications, such as microfluidic devices and biomedical systems.

A defining feature of PDMS is its tunable mechanical properties, allowing precise adjustments in stiffness by modifying the crosslinking agent ratio during polymerization. This adaptability enables the replication of tissue stiffness across a wide spectrum, from soft tissue to rigid tissue, making PDMS the preferred material for mimicking the ECM in tissue engineering [43]. Its biocompatibility supports the growth and proliferation of various cell types, including fibroblasts, without adverse reactions when studying cellular responses to mechanical stimuli. Furthermore, optical transparency facilitates real-time monitoring of cell cultures, enhancing the study of cell–matrix interactions under controlled conditions [44]. Interestingly, PDMS surface functionalization can be easily modified to improve adhesion, bioactivity, or functionality with a coating of extracellular matrix proteins, such as collagen and fibronectin, to promote cell attachment [45]. PDMS’s inherent hydrophobicity and low surface energy are advantageous for non-stick applications, such as microfluidic channels and lab-on-a-chip devices, and surface treatments can tailor its properties to enhance cell adhesion as needed [46]. These attributes collectively position PDMS as an ideal material for applications ranging from tissue engineering to inflammation models and advanced biomedical systems.

### 2.2. Hydrogel

Hydrogels, composed of hydrophilic polymer networks, are essential biomaterials for replicating the ECM because of their tunable mechanical properties, high water retention, and biofunctionalization capacity. These features make hydrogels particularly valuable for investigating cell–matrix interactions, especially in fibroblast studies [47]. The ECM is a complex network of proteins and polysaccharides that regulates critical cellular processes such as adhesion, migration, proliferation, and differentiation. Tissue-specific stiffness—a key ECM property—governs cellular behavior in both normal and pathological states [48]. The ability of hydrogels to mimic ECM stiffness enables recreating the mechanical and biochemical cues of native tissues, offering a platform for studying fibroblast responses under controlled conditions. In addition, hydrogels possess beneficial properties that are indispensable in biomedical applications. The high hydration capacity, supporting nutrient and waste transport, creates a physiologically relevant environment for cell culture. Hydrogels also exhibit tunable mechanical properties, allowing the stiffness to be tailored to mimic soft tissues (e.g., brain at ~0.1–1 kPa) or stiff tissues (e.g., bone at >1 GPa) [49]. Biocompatibility is another critical attribute, with natural hydrogels such as collagen and alginate supporting cell adhesion and growth, while synthetic variants such as polyethylene glycol (PEG) can be functionalized for enhanced cellular interactions. The porous structure of hydrogels facilitates molecular diffusion, which is critical for drug delivery and signaling studies. Moreover, bioactive functionalization enables the incorporation of growth factors, ECM proteins, or peptides to direct cell behavior [50].

Hydrogels are classified into natural, synthetic, and hybrid categories. Natural hydrogels, derived from ECM components, collagen, and hyaluronic acid, have excellent biocompatibility [51]. Synthetic hydrogels, including PEG and polyacrylamide, provide precise control over mechanical and degradation properties [52]. Hybrid hydrogels combine these advantages, balancing biocompatibility and tunability. The stiffness of hydrogels can be modified through crosslinking density, polymer composition, and gradient generation, enabling the study of dynamic mechanical changes in tissues. For instance, fibroblasts cultured on hydrogels with fibrotic tissue-like stiffness exhibit increased expression of activation markers, such as α-smooth muscle actin (α-SMA), illustrating stiffness-dependent transitions to myofibroblasts [53,54].

The polymer properties of hydrogels are fundamentally linked to their chemical structure, enabling their extensive functionality in biomedical applications. Hydrogels exhibit a remarkable capacity to absorb up to 99% of their weight in water, attributed to their hydrophilic polymer chains, thereby creating a hydrated environment that is optimal for cell culture and nutrient transport [55]. Their mechanical properties are highly adaptable, with stiffness modifiable through adjustments in polymer concentration, crosslinking density, or the incorporation of reinforcing materials. This tunability facilitates the replication of tissue-specific stiffness, ranging from soft brain tissue to rigid fibrotic tissues. The porous and permeable architecture of hydrogels supports efficient molecular diffusion, making them ideal for applications such as drug delivery and tissue engineering scaffolds. Natural hydrogels inherently promote cellular interactions, while synthetic hydrogels can be functionalized with bioactive molecules, including ECM proteins or growth factors, to enhance specific biological responses. Furthermore, hydrogels can be engineered for controlled degradability in response to environmental stimuli such as pH, temperature, or enzymatic activity, ensuring compatibility with targeted applications [56]. This unique combination of hydration, mechanical tunability, porosity, biocompatibility, and degradability positions hydrogels as exceptional materials for mimicking ECM stiffness, investigating cellular behaviors, and advancing innovations in tissue engineering, drug delivery, and inflammation studies.

In applications, hydrogels are prepared by dissolving polymers in a solvent, followed by crosslinking to form a gel. Atomic force microscopy (AFM) or rheometry measures the resulting stiffness [57]. Hydrogels provide platforms for fibroblast culture, morphological assessment, and differentiation studies. Additionally, hydrogels facilitate the investigation of inflammatory responses by exposing fibroblasts to cytokines like transforming growth factor-beta (TGF-β) and interleukin (IL)-1β, with gene and protein expression analyses revealing inflammatory markers such as tumor necrosis factor–α (TNF-α) and IL-6 [58].

PDMS and hydrogels are indispensable tools for replicating the mechanical properties of the ECM in vitro. Their tunable stiffness, biocompatibility, and versatility have enabled significant advances in studying cell–matrix interactions, particularly in the context of fibroblast behavior and inflammatory responses. By leveraging the unique properties of these materials, we have outlined a comparison of PDMS and hydrogels for ECM stiffness applications in Table 1. This comparison provides a foundation for researchers to develop innovative strategies in tissue engineering, disease modeling, and mechanobiology research. Future studies should focus on optimizing these materials and exploring hybrid systems to overcome their limitations and further expand their applications.

## 3. Surface Functionalization of Substrate Stiffness Materials

**Figure 1:**
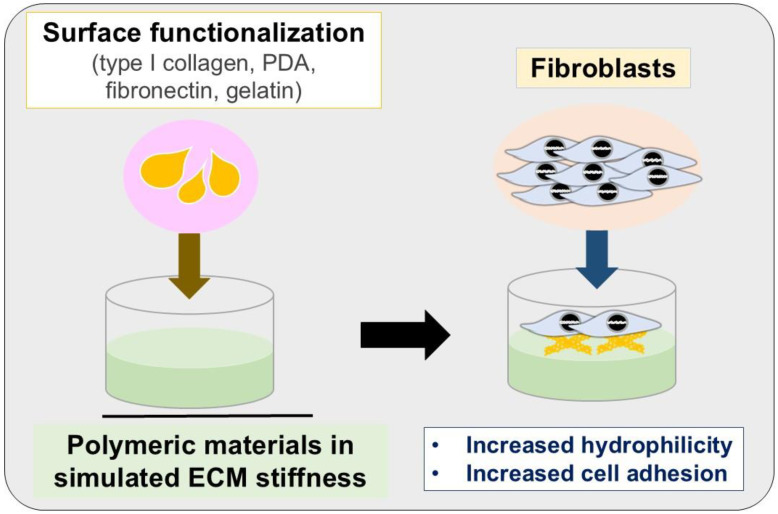
Surface functionalization on polymeric materials in simulated ECM stiffness for fibroblast cell adhesion. ECM, extracellular matrix; PDA, polydopamine.

Surface functionalization through coating materials is essential for improving the functionality of PDMS substrates in cell culture applications. Because of its inherent hydrophobicity, PDMS exhibits properties that inhibit protein adsorption and cell adhesion, often requiring the application of surface coatings to enhance its biocompatibility. Biochemical coatings, such as ECM proteins, including collagen, fibronectin, laminin, and gelatin, are widely employed to mimic the native cellular environment and promote adhesion (Figure 1). Functionalization with bioactive peptides, such as arginylglycylaspartic acid (RGD) motifs, further supports integrin-mediated cell attachment. Additionally, serum adsorption using fetal bovine serum (FBS) provides a cost-effective and non-specific method to enhance cell adhesion on PDMS surfaces [65,66].

Biochemical strategies and physical and chemical methods are often utilized to improve PDMS substrate properties. Oxygen plasma treatment is a commonly used technique to render the PDMS surface hydrophilic, facilitating the adsorption of ECM proteins or synthetic coatings like poly-L-lysine, which are particularly effective for neuronal culture applications [67]. In addition, synthetic polymers such as polyethylene glycol (PEG) are also employed to minimize non-specific interactions while supporting selective cell adhesion when combined with bioactive molecules [68]. These approaches collectively enable the customization of PDMS surfaces to address the specific requirements of diverse cell types.

Hydrogels, known for their hydrophilic properties, also benefit from similar coating strategies to enhance bioactivity. ECM protein coatings, including collagen, fibronectin, laminin, and Matrigel, replicate the structural and biochemical cues of native tissues, supporting cell adhesion and differentiation. Functionalization with RGD peptides improves the compatibility for synthetic hydrogels such as PEG or polyacrylamide. Advanced techniques, covalent crosslinking, and layer-by-layer coating ensure stable biomolecule attachment, further enhancing the hydrogel application in biological contexts. Combining PDMS and hydrogel substrates or incorporating gradients of coatings enables the creation of hybrid systems that support stiffness-guided cell migration and complex cellular behaviors [69].

### 3.1. Type I Collagen

Type I collagen, the most abundant ECM protein, is widely employed to enhance cell adhesion on PDMS and hydrogel substrates for its ability to mimic the native cellular microenvironment. RGD motifs facilitate integrin-mediated adhesion, promoting cell survival, proliferation, and differentiation. However, effective coating requires overcoming the inherent hydrophobicity of PDMS and ensuring hydrogel stability. Surface modification techniques, such as oxygen plasma treatment and polydopamine (PDA) coatings, are commonly used to improve surface wettability and provide reactive groups for collagen attachment. For instance, PDA-treated PDMS significantly enhances collagen adhesion and prevents detachment under dynamic conditions, making it suitable for long-term cell culture stability [65,70]. Similarly, covalent immobilization methods, such as carbodiimide chemistry, ensure there are stable collagen coatings resistant to delamination [71]. In hydrogel systems, collagen serves as a scaffold for 3D cultures, supporting cell migration and tissue development. Previous studies indicated that collagen coatings prepared using acetic acid solutions achieve homogeneous distribution and efficient cell attachment, even at low concentrations [71,72]. These advancements in collagen coating techniques not only improve biocompatibility, but also enable the development of hybrid systems for tissue engineering and mechanobiology applications.

### 3.2. Polydopamine

Polydopamine (PDA) coatings have emerged as a versatile and effective strategy for enhancing cell adhesion on PDMS and hydrogel substrates by addressing their inherent hydrophobicity and bioinert properties. Formed through the oxidative self-polymerization of dopamine in slightly alkaline conditions, PDA introduces catechol and amine groups that improve surface wettability and provide reactive sites for further functionalization. Previous studies have shown that PDA-coated PDMS significantly reduces water contact angles, facilitating protein adsorption and enhancing the adhesion and proliferation of human umbilical vein endothelial cells and mesenchymal stem cells compared to bare PDMS surfaces [66,73]. Plasma-assisted PDA coatings further improve adhesion strength and uniformity by increasing the grafting efficiency of dopamine, achieving up to threefold times higher shear adhesion strength than untreated PDMS. Additionally, PDA coatings such as collagen serve as an intermediary layer for ECM protein attachment, synergistically enhancing long-term cell adhesion and proliferation [65]. When applied to hydrogel systems, PDA significantly improves the stability of hydrogel constructs on PDMS substrates, enabling 3D culture models with prolonged functionality in tissue engineering applications [70,74]. These findings highlight the versatility of PDA coatings in modifying polymeric surfaces for various biomedical applications, making them a valuable tool in advancing tissue engineering and regenerative medicine.

### 3.3. Fibronectin

Fibronectin (FN) coatings have been widely utilized to enhance cell adhesion on polymer substrates, addressing the limitations posed by the substrates’ intrinsic hydrophobicity and bioinert properties. FN, a key glycoprotein of the ECM, promotes cell adhesion through the RGD motif, which interacts with integrins on cell membranes, triggering intracellular signaling pathways that enhance cell attachment, spreading, and proliferation. Previous studies have demonstrated that FN-coated PDMS surfaces significantly improve cell adhesion compared to uncoated surfaces, particularly for endothelial cells, which exhibit enhanced migration and spreading due to the increased surface hydrophilicity and energy provided by the FN layer [75,76]. Surface modification techniques such as physical adsorption, plasma treatment, and covalent immobilization have been employed to ensure there are effective FN coatings. Physical adsorption relies on weak electrostatic interactions, while plasma treatment introduces polar functional groups to improve the FN binding stability. Covalent immobilization using silanization methods ensures long-term coating stability under dynamic conditions [77,78]. When applied to hydrogel systems, FN coatings enhance the stability of hydrogel constructs on PDMS substrates, enabling the development of 3D culture models that mimic in vivo environments and support tissue engineering applications [79,80]. These findings underscore the versatility of FN coatings in modifying polymeric surfaces for biomedical applications.

### 3.4. Gelatin

Gelatin coatings have proven to be a versatile and effective strategy for enhancing cell adhesion by addressing the hydrophobicity and bioinert properties of polymer substrates. Derived from collagen, gelatin contains bioactive sequences that interact with integrins on cell surfaces, triggering intracellular signaling pathways that promote adhesion, proliferation, and migration. Previous studies have demonstrated that gelatin-coated PDMS significantly increases hydrophilicity, as evidenced by reduced water contact angles and improved cell attachment for various cell types, including human adipose-derived stem cells and endothelial cells [81,82]. Surface modification techniques, such as physical adsorption, plasma treatment, and covalent immobilization, are employed to enhance the stability and functionality of gelatin coatings. Plasma treatment introduces polar functional groups on PDMS, improving gelatin adsorption, while covalent immobilization using agents like EDC ensures long-term coating stability under dynamic conditions [83]. When applied to hydrogel systems, gelatin coatings improve the adhesion of hydrogel constructs to PDMS substrates, enabling the development of 3D culture models that mimic in vivo environments and support tissue engineering applications [84,85]. Interestingly, hyaluronic acid (HA)-gelatin hydrogel increases surface hydrophilicity and reduces bacterial adhesion [81]. These findings highlight the potential of gelatin coatings to modify polymeric surfaces for enhanced biocompatibility in biomedical applications.

## 4. Cellular Mechanotransduction Pathway Mediated by ECM Stiffness

Cellular mechanotransduction, the process by which cells convert mechanical cues from their environment into biochemical signals, plays a crucial role in regulating fibroblast inflammatory responses. ECM stiffness serves as a key biophysical factor influencing fibroblast activation, proliferation, and cytokine secretion. The alteration of ECM stiffness has been related to fibrosis, wound healing, and pathological conditions such as chronic inflammation. Fibroblasts sense and respond to ECM mechanical properties through integrin-mediated adhesion complexes and cytoskeletal remodeling, leading to downstream signaling cascades, including YAP/TAZ and TGF-β signaling that modulates inflammatory gene and protein expression. Therefore, how fibroblasts interpret ECM stiffness to drive inflammation through cellular mechanotransduction may provide critical insights into tissue repair mechanisms and disease progression.

### 4.1. Integrin-Mediated Adhesion Complexes and Cytoskeletal Remodeling

Integrins, which are transmembrane receptors that connect the ECM microenvironment and cytoskeleton, serve as primary mechanosensors. These receptors mediate the transmission of mechanical forces into cells and initiate signaling cascades that regulate cellular activities. Upon engaging with ECM ligands such as fibronectin and collagen, integrins cluster at focal adhesions, where they recruit adapter proteins and kinases, including focal adhesion kinase (FAK) and steroid receptor coactivator (Src). These components initiate downstream signaling pathways such as MAPK, RhoA, and PI3K/Akt, which influence fibroblast behavior under mechanical stress and the microenvironment [86]. The cytoskeleton plays a crucial role in transmitting tension from integrins to the nucleus. Actin filaments, intermediate filaments, and microtubules act as conduits for mechanical signals, forming a physical connection between the cell surface and the nucleus. This mechanical linkage enables the deformation of the nuclear architecture, which alters chromatin organization and gene expression [87]. A previous study found that mechanotransduction pathways are critical for cellular adaptation in tissues exposed to continuous mechanical forces, such as fibrotic microenvironments [15].

Rho-associated kinase (ROCK), a primary downstream effector of Rho, plays a pivotal role in driving cell contractility and mediating fibrotic pathologies [88,89]. By phosphorylating the myosin light chain and myosin light chain phosphatase, ROCK facilitates cellular force generation through actomyosin filament contraction. Recent studies revealed that ROCK has a stiffness-dependent role in the fibroblast-to-myofibroblast transition during pulmonary fibrosis, with inhibition shown to reduce the endothelial permeability associated with age-related intima stiffness [90].

Focal adhesion kinase (FAK) is a cytosolic nonreceptor tyrosine kinase, activated by integrin clustering, which plays a pivotal role in focal adhesion dynamics and Rho activity regulation [91,92]. Positioned upstream of the Ras–MAPK cascade, FAK integrates ECM signals from integrins to activate pathways governing cell adhesion, proliferation, motility, and survival [93]. FAK also mediates actomyosin force transmission to the ECM, making it a promising upstream target for disrupting cellular responses to matrix mechanical cues. In addition, matrix stiffness enhances FAK phosphorylation at Tyr397, enabling Src binding and complete FAK activation through further phosphorylation [94]. Stiffness-induced FAK activation contributes to pathologies. Constitutive phosphorylation of FAK is observed in fibrotic fibroblasts, where FAK signaling amplifies growth factor signaling and promotes the myofibroblast phenotype [95]. Furthermore, mechanically driven FAK-ERK pathways activate Rho GEF, particularly GEF-H1, highlighting FAK’s integral role in mechanotransduction and pathological cellular responses [96].

### 4.2. Yes-Associated Protein (YAP) and Mechanotransduction

YAP is a pivotal transcriptional co-regulator in the Hippo signaling pathway and a central player in mechanotransduction. YAP activity is regulated by mechanical stimuli, including ECM stiffness and cellular tension. Under low mechanical stress or in soft ECM environments, YAP is phosphorylated by upstream kinases such as LATS1/2, which retains it in the cytoplasm and marks it for degradation. Conversely, stiffer ECMs or increased cytoskeletal tension result in the dephosphorylation of YAP, facilitating YAP nuclear translocation where it interacts with transcription factors such as TEAD to promote the expression of genes related to cell proliferation, survival, and ECM synthesis [97]. Emerging studies are highlighting the role of YAP in maintaining tissue homeostasis and driving pathological changes. For example, fibroblasts cultured on stiffer matrices exhibit increased YAP nuclear localization, correlating with upregulated expression of fibrotic markers such as α-smooth muscle actin (α-SMA) and collagen type I. This mechanotransduction pathway underscores the significance of ECM stiffness in determining fibroblast fate and inflammatory responses [98].

Therefore, integrins and YAP exemplify the complexity and elegance of cellular mechanotransduction mechanisms that bridge mechanical stimuli with biochemical responses. Their roles in modulating fibroblast behavior and inflammation are pivotal for understanding tissue homeostasis and pathological conditions. The supporting scientific evidence not only illuminates their importance in mechanobiology but also positions these pathways as attractive targets for therapeutic interventions in fibrosis, dental tissue regeneration, and inflammation-driven diseases.

### 4.3. ECM Stiffness-Dependent Regulation of Transcription Factors and Signaling Pathways in Inflammatory Responses

ECM rigidity modulates the transcription factor nuclear factor κB (NF-κB), a pivotal mediator of inflammation. This phenomenon is consistent with the observation that aberrant connective tissue growth in tumor stroma and fibrosis resembles a dysregulated wound healing process, highlighting the intricate relationship between ECM mechanics and inflammatory pathways [99]. In vitro studies indicate that the Rho-dependent nuclear localization of NF-κB increases with substrate stiffness, driving the expression of inflammatory markers such as IL-1β, IL-8, and matrix metalloproteinases (MMP)-9 [99,100]. Moreover, ECM stiffness enhances NF-κB nuclear translocation in both nonmetastatic and metastatic breast cancer cells under TNF-α stimulation. Interestingly, NF-κB regulation by Rho is context-dependent, as Rho can negatively regulate NF-κB independently of ECM stiffness in the presence of other cues and TNF-α stimulation [101]. Although both NF-κB and YAP/TAZ activation are influenced by cell shape and ECM stiffness, NF-κB localization occurs rapidly within hours, whereas YAP/TAZ translocation requires days [102]. This highlights the distinct sensitivities of transcription factors to Rho-generated actomyosin forces.

In addition, stiffness-dependent integrin activation triggers mitogen-activated protein kinase (MAPK) signaling cascades, which regulate critical cellular processes such as survival, gene expression, and myofibroblast differentiation [103]. The stiffness-induced activation of MAPK signaling, particularly through the ERK pathway, is dependent on FAK, emphasizing the essential role of FAK in integrin-mediated mechanotransduction [104]. Furthermore, transforming growth factor-beta (TGF-β) drives the expression of connective tissue growth factor (CTGF) via Ras-ERK signaling, with emerging evidence indicating that CTGF expression is partially modulated by ECM stiffness [105]. The convergence of ECM stiffness, Rho activation, and MAPK signaling pathways holds significant implications for fibroblast-mediated inflammatory responses and their contribution to disease progression.

## 5. Fibroblasts as Key Regulators of Inflammation and Tissue Remodeling: From Homeostasis to Disease

**Figure 2:**
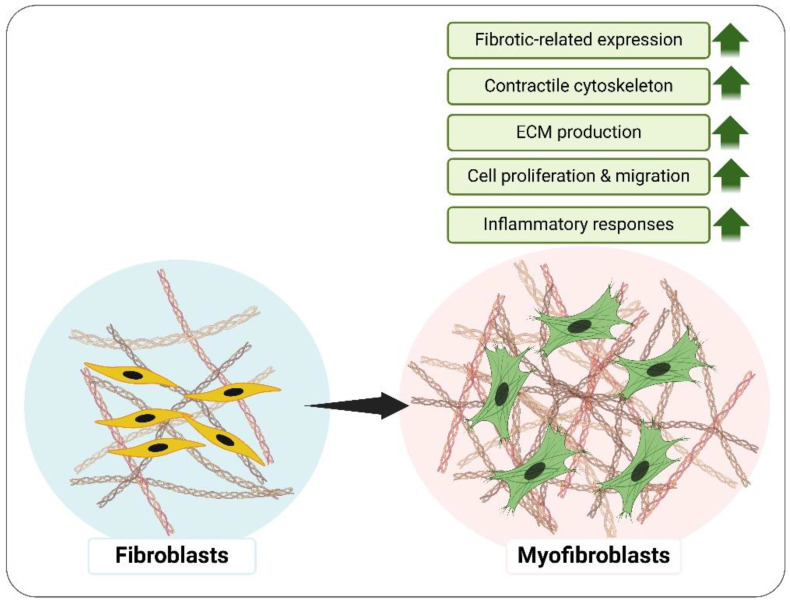
The inflammatory responses of myofibroblast differentiation via ECM stiffness. ECM, extracellular matrix.

Fibroblasts are pivotal cellular components in tissue homeostasis, repair, and inflammation. The mesenchymal-derived cells not only synthesize and maintain the ECM, but also actively participate in immune responses. During inflammation, fibroblasts exhibit phenotypic plasticity to interact with immune cells, secrete inflammatory mediators, and regulate tissue remodeling [106]. The functions of fibroblasts extend beyond their structural role in tissue support; they also act as sentinel cells, dynamically responding to microenvironmental signals and playing a critical role in regulating both acute and chronic inflammatory processes [107]. Under physiological conditions, fibroblasts maintain tissue integrity by producing ECM components such as collagen and fibronectin. However, during inflammation, fibroblasts are activated by cytokines such as TGF-β and IL-1β, transitioning to a pro-inflammatory state. This activation leads to the secretion of chemokines and cytokines, including IL-6 and TNF-α, which recruit immune cells to the site of injury or infection [108,109]. In chronic inflammatory conditions, such as rheumatoid arthritis and pulmonary fibrosis, fibroblasts sustain inflammation by creating a positive feedback loop of cytokine production and immune cell recruitment. Moreover, fibroblasts in inflamed tissues often acquire a myofibroblast phenotype, characterized by the expression of alpha-smooth muscle actin (α-SMA) and increased ECM deposition, which contributes to tissue stiffness and fibrosis [53,110]. Myofibroblasts are critical effector cells in fibrosis and wound healing, representing a heterogeneous population derived from multiple sources, including resident tissue cells, bone marrow progenitors, and immune cells, with the specific origin dependent on the pathological context [111]. Activated myofibroblasts, in contrast to the spindle-shaped quiescent fibroblasts, adopt a stellate morphology and display increased proliferation, migration, and fibroblast activation protein expression [112] (Figure 2). The primary distinction between myofibroblasts and fibroblasts lies in the ability to generate significant contractile forces, a function enabled by α-SMA expression and a robust contractile cytoskeleton [113]. In addition, fibroblast-mediated inflammation is also influenced by their spatial localization within tissues. In synovial tissues, for instance, fibroblasts form pannus-like structures that invade cartilage and bone, perpetuating inflammation and damage in diseases such as arthritis [114]. These cells also interact with endothelial cells to promote angiogenesis, and with immune cells to influence their activation and differentiation, further amplifying the inflammatory response [115].

In the oral environment, fibroblasts are abundant in the gingiva, periodontal ligament, and oral mucosa. In response to microbial invasion or tissue injury, fibroblasts in dental and oral tissues produce pro-inflammatory cytokines, such as IL-1β, TNF-α, and IL-6. These cytokines recruit immune cells, including neutrophils and macrophages, to the affected site, initiating an immune response. Additionally, oral fibroblasts secrete MMPs that degrade the ECM, facilitating immune cell infiltration and tissue remodeling [109]. In chronic periodontitis, fibroblasts sustain inflammation through a feedback loop involving cytokine production and interaction with immune cells [116]. Gingival fibroblasts, for example, interact with T cells and macrophages to perpetuate inflammation and mediate tissue destruction. These interactions are further exacerbated by bacterial components, such as lipopolysaccharides (LPS), which activate toll-like receptors (TLRs), including TLR-4 and TLR-2, on fibroblasts, amplifying inflammatory signaling pathways and leading to pro-inflammatory responses [117,118].

The dual role of fibroblasts in resolving acute inflammation and driving chronic inflammation highlights their potential as therapeutic targets. Strategies aimed at modulating fibroblast activation and cytokine production hold promise for mitigating inflammatory diseases and preventing fibrosis-associated complications.

## 6. The Role of ECM Stiffness in Fibroblast Activation and Inflammatory Diseases

Fibroblasts are essential players in tissue homeostasis and repair, but their dysregulation can lead to chronic inflammation and fibrosis. Emerging evidence highlights the role of ECM stiffness in regulating fibroblast behavior and inflammatory responses. By altering substrate stiffness, the mechanobiology of fibroblasts, including their cytokine production, activation, and signaling pathways, has gained more attention. The stiffness of the ECM is a critical determinant of fibroblast behavior, particularly in the context of inflammatory diseases. Fibroblasts, as primary producers of ECM components, are highly responsive to the mechanical properties of their environment, and changes in ECM stiffness can significantly impact their activation, proliferation, and secretion of pro-inflammatory mediators. Increased ECM stiffness—often characteristic of fibrotic tissues—enhances fibroblast activation and proliferation, contributing to the progression of inflammatory conditions [119].

Previous studies have demonstrated that fibroblasts cultured on hydrogels with stiffness levels mimicking fibrotic conditions of approximately 15–30 kPa exhibit increased nuclear translocation of YAP, a key regulator of cell proliferation and survival. This translocation is associated with the upregulated expression of fibrotic markers such as α-smooth muscle actin (α-SMA) and collagen type I, indicating a shift toward a myofibroblast phenotype that promotes excessive ECM production [102,120]. Conversely, fibroblasts on softer substrates of around 5 kPa display reduced contractility and lower levels of proliferation, highlighting the detrimental effects of increased stiffness on cellular behavior [121]. In addition to influencing fibroblast activation and proliferation, ECM stiffness modulates the secretion of crucial cytokines such as TGF-β and IL-1β. Elevated stiffness enhances the integrin signaling pathways that promote cell attachment and migration, leading to increased fibroblast activity in response to inflammatory stimuli. TGF-β further reinforces fibroblast activation and creates a positive feedback loop that exacerbates inflammation and fibrosis [122,123]. Moreover, ECM stiffness is associated with heightened secretion of the receptor activator of nuclear factor kappa-B ligand (RANKL), which plays a significant role in osteoclast differentiation, bone remodeling, and inflammation. Previous research indicated that fibroblasts on hydrogels engineered to mimic fibrotic tissue stiffness produce more RANKL compared to those on softer matrices. This elevated secretion contributes to a feedback loop in inflammatory conditions, where increased RANKL levels further stimulate osteoclast activity and promote inflammation [18]. Furthermore, a previous study reported that ECM stiffness induced the osteoclastogenic potential of the fibroblast cell line [124].

The mechanotransduction pathways activated by ECM stiffness are central to these processes. Integrins serve as mechanosensors that transmit mechanical signals from the ECM to intracellular signaling cascades, influencing gene expression related to inflammation and fibrosis. Specifically, YAP activation in response to increased stiffness has been linked to enhanced expression of fibrotic markers and cytokines [8]. Additionally, ECM stiffness influences the secretion of matrix metalloproteinases (MMPs), which are involved in ECM remodeling. Previous studies reported that fibroblasts on stiffer substrates show increased MMP expression, facilitating further remodeling of the ECM and contributing to the pathological state observed in inflammatory diseases [36,125]. Therefore, ECM stiffness significantly influences fibroblast activation, proliferation, and cytokine secretion through mechanotransduction pathways that alter cellular responses to mechanical stimuli. These changes perpetuate cycles of inflammation, fibrosis, cancer, and the tissue remodeling characteristic of chronic inflammatory diseases [126,127]. Investigating these mechanisms is essential for developing therapeutic strategies aimed at modulating ECM properties to mitigate inflammation and its associated effects on tissue remodeling. By elucidating the interplay between ECM stiffness, cytokine secretion, and fibroblast behavior, researchers can pave the way for innovative treatments targeting fibrotic diseases and chronic inflammatory conditions.

## 7. ECM Stiffness and Inflammatory Conditions Across Various Tissues

**Figure 3:**
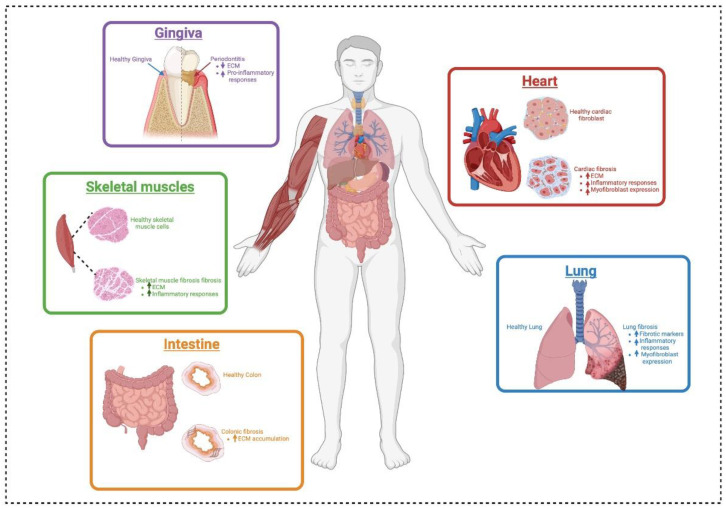
ECM stiffness-related inflammatory tissue in multiple organ systems. ECM, extracellular matrix.

Tissue fibrosis is a pathological condition characterized by excessive ECM deposition, leading to increased tissue stiffness and impaired organ function. This phenomenon occurs across multiple organ systems, including the heart, lungs, skin, intestines, and skeletal muscles (Figure 3). Fibrosis is primarily driven by fibroblast activation and differentiation into myofibroblasts, which secrete profibrotic cytokines and contribute to ECM remodeling. The mechanical properties of the ECM, particularly stiffness, play a crucial role in regulating fibroblast behavior, including fibroblast activation, proliferation, and cytokine secretion through mechanotransduction pathways, ultimately exacerbating fibrotic progression and inflammation (see Table 2).

### 7.1. Cardiac Fibrosis

In cardiac tissue, increased ECM stiffness is associated with heart diseases such as hypertension and myocardial infarction. Fibroblasts in the heart respond to mechanical cues by becoming activated and differentiating into myofibroblasts, which contribute to excessive ECM deposition and cardiac remodeling [17]. Elevated stiffness in the cardiac ECM promotes the secretion of pro-inflammatory cytokines, leading to a positive feedback loop that exacerbates inflammation and fibrosis via the mechanotransduction pathway [128,129,130,131]. Furthermore, increased ECM stiffness induced cell proliferation and ECM production via FAK and actin polymerization in cardiac fibroblasts [132]. Studying this process is key to understanding heart failure mechanisms and developing targeted therapies.

### 7.2. Pulmonary Fibrosis

Pulmonary fibrosis is characterized by excessive deposition of collagen and other ECM components, resulting in increased stiffness of lung tissue [133]. Previous studies demonstrated that fibroblasts exposed to stiffer substrates exhibit enhanced secretion of cytokines such as IL-1β and RANKL. These cytokines contribute to the recruitment of immune cells and further promote inflammation within the lung microenvironment [18]. Furthermore, fibroblast activation induced by a stiff matrix is involved in myofibroblast expression of the pathophysiology of pulmonary fibrosis [134]. The mechanotransduction pathways activated by ECM stiffness play a central role in this process, highlighting the importance of targeting ECM properties for therapeutic interventions in pulmonary diseases.

### 7.3. Skin Fibrosis

Skin fibrosis involves the excessive accumulation of ECM components, leading to increased stiffness that disrupts normal skin function [119]. Previous research indicated that fibroblasts in fibrotic skin exhibit heightened activation and proliferation due to increased stiffness. The mechanical signals from the stiffened ECM activate YAP signaling pathways, resulting in the upregulation of profibrotic cytokines and further promoting fibroblast activation. This creates a positive feedback loop that perpetuates skin fibrosis [135].

### 7.4. Intestinal Fibrosis

In intestinal fibrosis, increased ECM stiffness is associated with chronic inflammatory conditions such as Crohn’s disease [136]. The stiffened ECM influences fibroblast behavior by enhancing the cell proliferative capacity and secretion of inflammatory mediators [137]. Elevated levels of TGF-β in response to increased stiffness further exacerbate inflammation and promote fibrotic processes in the intestinal tissue [138].

### 7.5. Skeletal Muscle Fibrosis

Skeletal muscle fibrosis results from various injuries or chronic conditions that lead to increased ECM deposition and stiffness [139]. Fibroblasts within skeletal muscle respond to mechanical changes by secreting cytokines that promote inflammation and inhibit muscle regeneration [140]. A recent study suggests that the stiff ECM promotes the myogenic-to-fibrogenic conversion of muscle stem cells via an inflammatory TNF-related apoptosis-inducing ligand pathway, which could lead to overt ECM production and fibrosis, causing the functional limitation of the affected muscles [140,141]. Although the exact mechanisms by which collagen modulates myalgia are not fully understood, it is hypothesized that it may enhance ECM remodeling by promoting muscle regeneration and preventing fibrosis [142,143]. Studying the interplay between mechanical cues from the ECM stiffness and fibroblast activity is crucial for understanding muscle healing processes.

### 7.6. Gingival and Periodontal Disease

In periodontal disease, bacterial biofilm induces periodontal tissue, leading to inflammatory gingivitis and periodontitis with loss of attachments. Altered ECM stiffness affects fibroblast function within gingival tissues. Decreased stiffness promotes fibroblast activation and secretion of inflammatory cytokines, such as IL-1β and PGE_2_, through NF-κB and YAP signaling pathways that contribute to tissue destruction [35]. Furthermore, a previous study revealed that ECM stiffness regulated cell proliferation and osteogenic differentiation of periodontal ligament stem cells [144]. The mechanical properties of the periodontal ECM are critical for maintaining periodontal health; thus, understanding these dynamics can inform therapeutic strategies for managing periodontal diseases.

**Table 2:** Regulation of inflammatory responses via extracellular matrix stiffness in various tissues.

**Tissue Mimetic**	**Cell Type**	**Substrate Material**	**Coating**	**Results**	Refs.
Heart fibrosis	Cardiac fibroblasts	poly(ethylene glycol) (PEG) hydrogels	CRGDS peptide	Substrate stiffness regulates fibrotic gene and inflammatory response via NF-κB pathway	[130]
Heart fibrosis	Cardiac fibroblasts	polyacrylamide gels	Type I Collagen	Substrate stiffness influences the releasing of IL-6, IL-11, sIL-6R	[131]
Lung fibrosis	Lung fibroblasts	polyacrylamide gels	Type I Collagen	Substrate stiffness controls fibrotic marker and RANKL expression	[18]
Intestinal fibrosis	Colonic myofibroblasts	polyacrylamide gels	Type I Collagen	Substrate stiffness modulates profibrotic response of colonic myofibroblasts	[145]
Gingival tissue	Gingival fibroblasts	PDMS	Type I Collagen	Substrate stiffness controls pro-inflammatory cytokines and ECM production	[35]
Skeleton muscle fibrosis	Muscle stem cell	PDMS	Fibronectin	Substrate stiffness regulates TNF-related apoptosis-inducing ligand (TRAIL) pathway	[140]

ECM, extracellular matrix; PDMS, polydimethylsiloxane; NF-κB, nuclear factor-kappa B; IL, interleukin; RANKL, receptor activator of nuclear factor kappa-Β ligand; TNF, tumor necrosis factor.

ECM stiffness serves as a critical regulator of fibroblast behavior across various tissues affected by inflammatory conditions. ECM stiffness not only enhances fibroblast activation and proliferation, but also modulates the secretion of key cytokines involved in inflammation and tissue remodeling. Additionally, fibroblasts from different tissues exhibit unique phenotypic and functional characteristics. For example, lung fibroblasts, which play a critical role in maintaining alveolar structure, are often more sensitive to mechanical cues and may undergo differentiation at lower stiffness levels [146]. In contrast, dermal fibroblasts, which are involved in wound healing and scar formation, typically require higher stiffness to trigger myofibroblast transformation [147]. Interestingly, epigenetic regulatory factors involved in ECM stiffness-induced tissue fibrosis or pathology, including DNA methylation and Histone modification, which further contribute to sustained fibroblast activation and pathological fibrosis [148]. Therefore, understanding these mechanisms is essential for developing therapeutic strategies aimed at modulating ECM properties to mitigate inflammation and its associated effects on tissue pathology. Future research should focus on elucidating the specific mechanotransduction pathways involved in these processes to pave the way for innovative treatments targeting fibrotic diseases.

## 8. Targeting Fibroblast-Mediated Inflammation for Biomedical Applications

**Figure 4:**
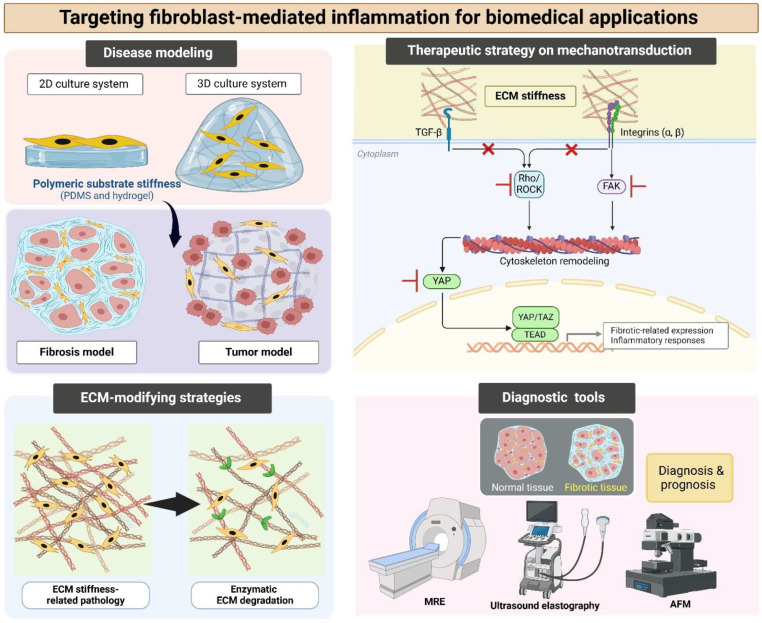
Targeting fibroblast-mediated inflammation for biomedical applications, including disease modeling, therapeutic strategy on mechanotransduction mechanism, ECM-modifying strategies, and diagnostic tools such as MRE, ultrasound elastography, and AFM. ECM, extracellular matrix; PDMS, polydimethylsiloxane; TGF-β, transforming growth factor-beta; ROCK, Rho-associated kinase; FAK, focal adhesion kinase; YAP, Yes-associated protein; TAZ, transcriptional coactivator with PDZ-binding motif; MRE, magnetic resonance elastography; AFM, atomic force microscopy.

The alteration of ECM stiffness is implicated in pathologies such as fibrosis, chronic inflammation, and cancer. Increased ECM stiffness drives disease progression by influencing cellular behavior, mechanotransduction pathways, and tissue remodeling, underlying the mechanobiological mechanisms involved in developing new therapies. Mechanobiology, the study of how physical forces and mechanical properties affect cellular function, offers promising strategies for biomedical application by targeting ECM stiffness and associated pathways. With advancements in biomaterials, mechanosensitive drug delivery, and tissue engineering, mechanobiology-based approaches hold significant potential for disease treatment, emphasizing the biomedical applications of ECM tissue stiffness, its role in pathology, and the potential of mechanobiological strategies in therapeutic development (Figure 4).

### 8.1. Disease Modeling Applications

ECM stiffness has garnered attention for its role in disease progression and pathophysiology [102,149]. Abnormal ECM stiffness is associated with diseases such as cancer, fibrosis, and cardiovascular disorders, in which tissue mechanics influence cellular functions, including proliferation, migration, differentiation, and inflammatory response [150]. Understanding the mechanobiological interactions is crucial for developing physiologically relevant disease models [149]. Recent advancements in biomedical research have led to the development of in vitro and in vivo models incorporating ECM stiffness to mimic disease conditions, enabling us to study disease mechanisms and screen potential therapeutics. By integrating biomaterials, two- and three-dimensional culture systems, and microfluidic technologies, we can create more accurate representations of pathological microenvironments [151,152,153]. These models allow for investigations into the impact of matrix stiffness on cancer progression, immune cell recruitment, and responses to immunotherapy [127]. This approach supports understanding and treating complex diseases and developing personalized medicine strategies.

**(1)**  **Two-dimensional and three-dimensional tumor models**

Engineered hydrogels with tunable stiffness have been used to mimic the TME and study cancer cell behavior. These models provide insights into how ECM stiffness influences drug resistance and metastatic potential [154]. Additionally, brain-on-a-chip models have been introduced. Stiffness-tunable hydrogels have been used to model the brain ECM and study the effects of stiffness on neuronal and glial cell behavior. These models provide insights into the role of ECM mechanics in neurodegenerative diseases and brain tumors [155].

**(2)**  **In vitro model for fibrosis tissue**

Stiffness-tunable substrates have been used to study fibroblast activation and ECM remodeling in fibrotic diseases. These models identify key mechanosensitive pathways, such as TGF-β signaling, that contribute to fibrosis [156,157]. In addition, engineered heart tissues, via biomaterials with controlled stiffness, have been used to model cardiac fibrosis and study the effects of ECM stiffness on cardiomyocyte function [158]. These models provide insights into the role of ECM mechanics in heart failure.

### 8.2. Therapeutic Strategy for Fibroblast Inflammation in Mechanotransduction Mechanisms

Targeting mechanotransduction pathways in fibroblasts has emerged as a promising therapeutic strategy to treat diseases driven by aberrant fibroblast activation, such as fibrosis, cancer, and chronic inflammatory disorders. Mechanotransduction in fibroblasts involves a complex network of signaling pathways that respond to changes in ECM stiffness and mechanical forces. These pathways include integrin-mediated signaling, Rho/ROCK signaling, cytoskeletal remodeling, YAP/TAZ signaling, and TGF-β signaling. Dysregulation of these pathways contributes to pathological fibroblast activation, leading to excessive ECM deposition, tissue stiffening, and organ dysfunction. The concept of mechanobiology, which explores how mechanical forces influence cellular behavior, provides a framework for developing novel therapeutic interventions to prevent and treat fibroblast-related pathologies. Therefore, targeting these pathways can help prevent and treat fibroblast inflammation-related pathologies, as follows:

**(1)**  **Targeting integrins**

Targeting integrins, which are transmembrane receptors mediating cell–ECM interactions, has emerged as a promising therapeutic strategy for managing fibrotic diseases. The inhibition of specific integrins has shown potential in both preclinical and clinical settings. Cilengitide, a cyclic peptide inhibitor of αvβ3 and αvβ5 integrins, demonstrates antifibrotic and antitumor effects by disrupting fibroblast–ECM interactions [159]. Similarly, ATN-161 targeted α5β1 integrin with antifibrotic and antitumor activity in preclinical models by inhibiting fibroblast activation. Additionally, ATN-161 decreased the expression of MMP-8 and -9 through α5β1 integrin [160]. Recently, Bexotegrast has gained attention as a dual inhibitor of αvβ6 and αvβ1 integrins overexpressed in fibrotic tissues. It shows promise in slowing or halting disease progression in idiopathic pulmonary fibrosis (IPF), which is supported by strong preclinical data and early-phase clinical trials [161]. Additionally, Pliant Therapeutics has developed compounds targeting αvβ6 integrin for treating various forms of fibrosis, including IPF [162]. These advancements highlight the therapeutic potential of targeting specific integrin subtypes to modulate the mechanotransduction pathways involved in disease pathology.

**(2)**  **Targeting the FAK pathway**

FAK is a crucial mediator of integrin signaling, playing significant roles in fibroblast survival, migration, and activation. Small-molecule inhibitors of FAK have demonstrated efficacy in preclinical models by disrupting fibroblast activation and reducing ECM production [163,164]. This approach offers the following dual benefits: it can reduce fibrosis while limiting tumor progression.

Recent advances have highlighted the potential of combining FAK inhibitors with other therapies to enhance their effectiveness. For instance, combining FAK inhibitors with chemotherapy or immunotherapy may improve outcomes against drug-resistant cancers. Additionally, emerging research suggests that targeting nuclear FAK could alter the tumor microenvironment by promoting immune surveillance and reducing angiogenesis [165]. Some drug companies are developing potent FAK inhibitors for both cancer and fibrosis treatment.

Furthermore, new strategies aim to overcome resistance to single-agent therapies by co-targeting pathways such as ERK5 alongside FAK inhibition in lung cancer patients with KRAS mutations [166]. Overall, targeting the FAK pathway presents an innovative approach to modulating the mechanotransduction processes involved in disease pathology.

**(3)**  **Targeting the Rho/ROCK pathway**

The Rho/ROCK pathway plays a crucial role in regulating the cytoskeletal dynamics essential for fibroblast contractility and activation. Fasudil has demonstrated antifibrotic effects in various tissues by reducing fibroblast contractility and ECM production [167]. Ripasudil is another ROCK inhibitor being explored for treating fibrotic diseases and ocular disorders [168]. Recent studies have highlighted the potential of selective ROCK2 inhibitors, such as GV101, in treating liver fibrosis by targeting multiple pathways simultaneously [169]. Clinical trials are ongoing to evaluate ROCK inhibitors across various conditions; while AT13148 faced challenges as a dual ROCK-AKT inhibitor for solid tumors, ongoing research has focused on optimizing dosing strategies and combining these inhibitors with other therapies to enhance their effectiveness against resistant tumors or diseases such as pulmonary fibrosis [170].

**(4)**  **Targeting YAP/TAZ signaling**

YAP and TAZ are transcriptional coactivators that respond to mechanical cues and promote fibroblast activation; their aberrant activation is implicated in both fibrosis and cancer. Verteporfin, an FDA-approved drug, inhibits YAP/TAZ nuclear localization and has demonstrated antifibrotic effects in animal models [171]. Compounds targeting the interaction between YAP/TAZ and TEAD transcription factors are under development, offering a novel approach to suppressing fibroblast activation [172]. Recent studies highlight the complex roles of YAP/TAZ in regulating inflammation through interactions with innate immune cells [173]. Additionally, Golgi-associated TAZ has been identified as a potential molecular target for treating tumor progression and chronic inflammation by modulating vesicle trafficking independently of the Hippo pathway [174]. These findings underscore the potential of targeting YAP/TAZ signaling for innovative therapeutic interventions across various diseases.

**(5)**  **Targeting TGF-β signaling**

TGF-β plays a pivotal role in regulating fibrosis by promoting fibroblast differentiation into myofibroblasts and enhancing ECM production. Targeting the TGF-β signaling pathway has shown promise in preclinical and clinical settings for treating fibrotic diseases. Galunisertib (LY2157299), a selective TGF-β receptor I kinase inhibitor, has demonstrated efficacy in reducing fibrosis in both preclinical models and early-phase clinical trials [175]. Additionally, anti-TGF-β antibodies have been developed to block TGF-β signaling, preventing fibroblast activation and reducing tissue fibrosis [176]. Recent research has highlighted the complex roles of TGF-β in cancer development, acting as a tumor suppressor early on and later promoting tumor progression [177]. In the context of inflammation and immunity, TGF-β modulates immune responses within the tumor microenvironment [177,178], emphasizing its potential as a therapeutic target across various diseases.

### 8.3. ECM-Modifying Strategies:

Modifying ECM stiffness through enzymatic degradation, biomaterial engineering, and hydrogel-based therapies has emerged as a promising strategy for treating ECM stiffness-related diseases.

**(1)**  **Enzymatic ECM degradation**

Enzymes such as collagenase or hyaluronidase can reduce ECM stiffness by degrading ECM components. This approach has been shown to suppress fibroblast activation, which is beneficial in reducing fibrotic responses [179,180]. However, the application of these enzymes must be carefully controlled to avoid excessive degradation that could lead to unintended consequences.

**(2)**  **Biomaterials with tunable stiffness**

Engineered biomaterials with adjustable mechanical properties offer a promising tool for studying fibroblast behavior under different conditions of stiffness. These materials can mimic pathological stiffness or counteract it by providing a more physiological environment for cell growth [127]. This approach allows researchers to develop therapies tailored to specific diseases where ECM dysregulation plays a critical role.

**(3)**  **Small molecules and increased ECM stiffness**

Interestingly, some studies have found that increased ECM stiffness can inhibit inflammatory responses in certain types of fibroblasts [127]. For instance, increased collagen and hyaluronic acid (HA) levels help maintain periodontal tissue health by regulating fibroblast proliferation and inflammatory responses [181]. This observation highlights the potential benefits of modulating ECM composition using small molecules or other agents that enhance mechanical properties.

**(4)**  **Hydrogels in therapeutic applications**

Hydrogels have emerged as versatile tools for modulating tissue mechanics, owing to their ability to adjust their mechanical properties according to the needs of specific applications. Hydrogels are particularly useful in treating conditions such as osteoarthritis because they can withstand pressure while providing lubrication [182]. The use of HA hydrogels has shown promise in maintaining higher HA concentrations over longer periods compared to traditional HA injections [183].

In animal models, HA hydrogel has demonstrated anti-inflammatory effects by reducing the NF-kB expression associated with inflammation and serving as a carrier for therapeutic exosomes derived from dental pulp stem cells [184]. Additionally, tilapia type I gelatin/HA hydrogel has been shown to decrease the release of pro-inflammatory cytokines such as MMP-9, IL-1β, IL-6, and TNF-α [185,186].

### 8.4. Diagnostic and Prognostic Tools for ECM Stiffness-Related Diseases

Accurate diagnosis and prognosis of ECM stiffness-related diseases are essential for effective treatment and management. Advances in imaging techniques and biomarker discovery have provided effective tools to assess tissue stiffness and predict disease progression. Therefore, non-invasive imaging methods are essential tools for the evaluation of tissue stiffness, enabling the early diagnosis and monitoring of disease progression. These techniques provide real-time, quantitative measurements of ECM mechanical properties.

**(1)**  **Magnetic resonance elastography (MRE)**

MRE is a non-invasive imaging technique that measures tissue stiffness by propagating mechanical waves through the tissues and analyzing their propagation patterns. MRE has been extensively used to assess liver fibrosis, where increased tissue stiffness correlates with disease severity [187]. Recent studies have also applied MRE to evaluate ECM stiffness in cancer and inflammatory diseases, providing insights into disease progression and treatment response [188,189].

**(2)**  **Ultrasound elastography**

Ultrasound elastography is another non-invasive imaging technique that measures tissue stiffness by analyzing the deformation of tissues under applied pressure [190]. It includes several subtechniques, one of which is strain elastography, which involves applying external pressure to measure strain in tissues. Strain ratio calculations can be used without knowing the applied force, making them practical for clinical use [23,191] and shear wave elastography, which uses acoustic radiation force impulses to generate shear waves within tissues. The speed of these waves is directly related to tissue stiffness and can be quantitatively measured as Young’s modulus or shear wave speed [23,192]. Ultrasound elastography is commonly used to diagnose liver fibrosis and breast cancer, where increased tissue stiffness is associated with tumor aggressiveness and poor prognosis [193].

**(3)**  **Atomic force microscopy (AFM)**

AFM is a high-resolution technique that measures tissue stiffness at the nanoscale level. AFM has been utilized to study ECM stiffness in fibrotic tissues and tumors, providing detailed insights into the mechanical properties of diseased tissues [194]. Its ability to probe local mechanical properties makes it invaluable for understanding cellular interactions with their microenvironment.

## 9. Conclusions and Future Prospects

**Figure 5:**
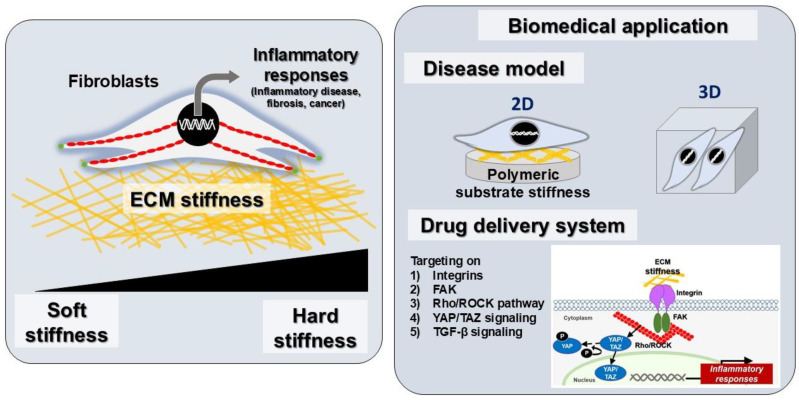
Extracellular matrix stiffness mechanotransduction and mechanobiological response-driven strategies for biomedical applications targeting fibroblast inflammation. ECM, extracellular matrix; FAK, focal adhesion kinase; ROCK, Rho-associated kinase; YAP, Yes-associated protein; TGF-β, transforming growth factor-beta.

This review underscores the pivotal role of ECM stiffness in modulating fibroblast behavior and driving inflammatory responses. Recent advancements in engineered biomaterials such as PDMS and hydrogels have provided versatile platforms for replicating tissue-specific stiffness. These tools enable precise control over cell–matrix interactions and facilitate the study of mechanotransduction pathways, including integrin signaling and YAP/TAZ activation. The strategies for modifying ECM properties, including tunable biomaterials and hydrogel-based therapies, hold significant promise for applications in tissue engineering, drug delivery, and anti-inflammatory treatments. However, further research is needed to develop precise biophysical models that accurately replicate complex tissue environments. Scalable production of tunable biomaterials will be crucial for advancing translational research into large-scale applications in regenerative medicine. Furthermore, exploring hybrid systems that combine different materials can enhance platform functionality. Investigating mechanotransduction pathways, including TGF-β signaling, will offer deeper insights into fibroblast responses to ECM stiffness (Figure 5). Integrating advanced imaging techniques with biomarker discovery will improve the diagnostic tools for diseases related to ECM stiffness. Ultimately, bridging laboratory findings with clinical applications is essential for developing effective therapies that mitigate chronic inflammation and fibrosis by modulating ECM properties.

## Figures and Tables

**Table 1:** Comparison of PDMS and hydrogel as materials for extracellular matrix (ECM) stiffness.

Property	PDMS	Hydrogel	Refs.
Stiffness range	5 kPa–10 MPa	0.1–100 kPa (cell culture plate)	[26,41,42]
Tunability	Stiffness is adjusted by varying crosslinker ratio and curing conditions	Stiffness is tuned by adjusting polymer concentration and crosslinking density	[33,59]
Biocompatibility	Generally biocompatible but requires surface functionalization for cell adhesion	Naturally biocompatible and can incorporate bioactive molecules (e.g., peptides, growth factors)	[9,49,60]
Degradability	Non-degradable	Degradable or non-degradable depending on polymer composition and crosslinking	[30,61]
Hydration	Low (hydrophobic)	High (hydrophilic)	[47,62]
Applications	Microfluidics, cell mechanobiology, and static ECM stiffness models	Dynamic ECM stiffness models, tissue engineering, and drug delivery	[56,63,64]

## Data Availability

Not applicable.
